# The Plant Sucrose Synthase Gene Family: Multi-Level Regulatory Networks and Functional Diversification in Plants

**DOI:** 10.3390/biom16050627

**Published:** 2026-04-23

**Authors:** Jiayao Lyu, Zongsuo Liang, Chenlu Zhang, Shuang Liu

**Affiliations:** 1 School of Biological Science and Engineering, Shaanxi University of Technology, Hanzhong 723000, China; lujiayao@snut.edu.cn; 2 College of Life Science, Zhejiang Sci-Tech University, Xuelin Road, Xiasha District, Hangzhou 310018, China; liangzs@ms.iswc.ac.cn

**Keywords:** sucrose synthase, SUS gene family, organ-specific expression, subcellular localization, phylogeny, regulatory mechanisms, transcriptional regulation, phosphorylation, protein interactions

## Abstract

Sucrose synthase (SUS) is a key enzyme in plant carbon metabolism, catalyzing the reversible interconversion between sucrose + uridine diphosphate (UDP) and UDP-glucose (UDP-Glc) + fructose. It plays a central role in carbon flux allocation, cell wall and starch synthesis, as well as plant development and stress responses. SUS is encoded by a multigene family whose members exhibit significant functional diversification and expression specificity across species, tissues, and subcellular compartments. This review systematically summarizes the physiological functions of SUS in source–sink regulation, seed filling, and rapidly growing tissues; describes the organ-specific expression patterns and diverse subcellular localizations of different isoenzymes in *Arabidopsis* and major crops; and elucidates the phylogenetic pattern of the SUS gene family into three evolutionary clades—SUS I, SUS II, and SUS III—based on a comparative analysis of selected angiosperm species. Furthermore, it integrates the multi-level regulatory mechanisms of SUS, including transcriptional and post-transcriptional regulation, as well as the dynamic control of enzyme activity, stability, and subcellular localization through post-translational modifications such as phosphorylation and ubiquitination and protein interactions. Finally, this study identifies gaps in current research regarding ubiquitination mechanisms, metabolic network integration, and crop applications. It envisions SUS-centered molecular breeding strategies, informed by integrative regulatory genomics, multi-omics, and genome editing, to redirect crop carbon fluxes and thereby enhance yield, improve quality traits, and increase stress tolerance.

## 1. Introduction

**Figure 1:**
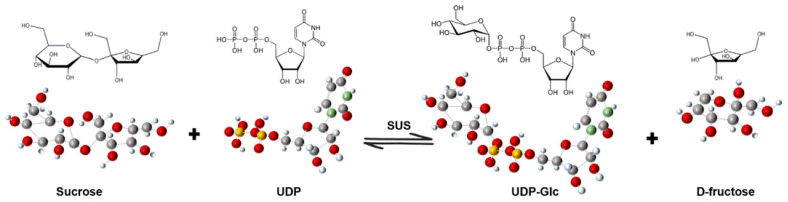
Reversible reaction catalyzed by sucrose synthase (SUS). SUS catalyzes the reversible interconversion between sucrose + UDP and UDP-Glc + fructose. This reaction underpins the central role of SUS in carbon partitioning, energy metabolism, and the provision of UDP-Glc for cell wall and starch biosynthesis.

Sucrose serves as the primary form for transporting photosynthetic products out of source tissues, acting as the central hub for carbohydrate transport and metabolism in plants [1,2,3]. In plants, sucrose cleavage can be mediated mainly by two enzyme systems, invertase and sucrose synthase (SUS) [4], whereas sucrose synthesis is classically mediated by sucrose phosphate synthase (SPS), which catalyzes the formation of sucrose-6-phosphate [5]. Invertase catalyzes the irreversible hydrolysis of sucrose into glucose and fructose, whereas SUS catalyzes the reversible interconversion between sucrose + UDP and UDP-glucose (UDP-Glc) + fructose [4,6]. These two pathways differ not only in reaction properties, but also in their physiological implications for carbon partitioning, metabolic efficiency, and sink activity [4]. In general, invertase is often associated with irreversible sucrose hydrolysis and hexose accumulation, whereas SUS is more closely linked to UDP-Glc supply for biosynthetic metabolism and is frequently important in storage tissues and under conditions in which energy conservation is advantageous [6,7]. Sucrose synthase is a key sugar transferase widely distributed throughout the plant kingdom. It is encoded by a small multigene family and functions in different tissues and developmental stages [6,8,9]. SUS catalyzes the reversible interconversion between sucrose + uridine diphosphate (UDP) and UDP-Glc + fructose (Figure 1) [10]. Through this reaction, it occupies a central position in carbon partitioning, cell wall biosynthesis, starch accumulation, signal transduction, and plant responses to diverse environmental stresses [11,12,13,14].

The catalytic direction of SUS is strongly influenced by the cellular microenvironment and shows a pronounced pH dependence. In general, the sucrose-synthesizing reaction is favored under neutral-to-alkaline conditions, whereas the sucrose-cleaving reaction is favored under mildly acidic-to-neutral conditions. Around pH 7.5, which lies near the boundary of these ranges, either reaction direction may occur depending on local substrate/product ratios and the cellular microenvironment [6,10,15,16,17]. Although the reaction is in principle reversible, current evidence indicates that SUS operates predominantly in the sucrose-degrading direction in vivo [11,18,19]. By continuously supplying UDP-Glc as a substrate for cell wall formation, starch biosynthesis, and multiple other metabolic pathways, SUS is indispensable for maintaining dynamic carbon flux homeostasis, securing a stable energy supply and supporting tissue growth [12,20,21,22].

Beyond its pH dependence and reversible catalytic behavior, structural studies have provided additional insight into the molecular characteristics of SUS. The crystal structure of *Arabidopsis thaliana* sucrose synthase 1 (*AtSUS1*) revealed that the enzyme forms a homotetramer and contains an N-terminal regulatory region together with a C-terminal catalytic GT-B glycosyltransferase region [23]. However, SUS proteins are not restricted to homotetrameric assembly. In maize, different SUS isoforms have been reported to adopt distinct oligomeric configurations, with SUS2 occurring predominantly as a hetero-oligomer with SUS1, whereas SUS-SH1 forms mainly homo-oligomers [24]. These different protein configurations are important because they are associated with differences in intracellular distribution, membrane association, and phosphorylation status, and may thereby contribute to isoform-specific differences in catalytic behavior and subcellular localization [24]. Within the N-terminal portion, a cellular targeting domain and an ENOD40 peptide-binding domain have been proposed, whereas the substrates bind in the interdomain cleft of the GT-B catalytic region [23]. These structural features indicate that SUS function depends not only on its catalytic activity, but also on its ability to associate with specific cellular targets. Consistent with this view, SUS has been reported to interact with membranes, cytoskeletal actin, and in some species or tissues, cell wall biosynthetic machinery or metabolically coupled enzymes [13,25]. However, the strength and stability of these interactions appear to be species- and context-dependent, and in *Arabidopsis*, the current evidence mainly supports a predominantly cytosolic role rather than a constitutive association with the cellulose synthase complex [26,27].

## 2. SUS as a Central Regulatory Hub

**Figure 2:**
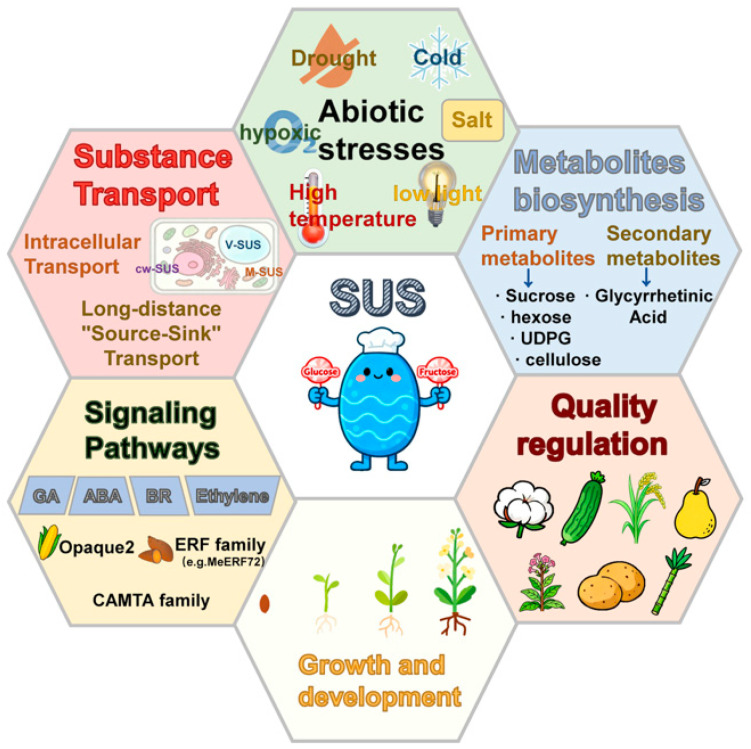
Overview of the major biological functions of sucrose synthase (SUS) in plants. This schematic summarizes the major roles of SUS in source–sink carbon partitioning, seed filling, fruit and fiber development, cell wall biosynthesis, starch accumulation, stress adaptation, and crop quality formation. Together, these functions position SUS as a central metabolic hub linking plant growth, development, and environmental responses.

At the whole-plant level, sucrose synthase occupies a central position in the control of carbon fluxes, particularly by acting as a key bridge for carbon partitioning between source and sink tissues [18,26]. By catalyzing the reversible interconversion between sucrose + UDP and UDP-Glc + fructose, SUS facilitates the transfer and utilization of carbohydrates among different organs, thereby not only providing a stable carbon and substrate supply for energy metabolism, but also directly supporting the accumulation of structural components required for cell expansion [28,29]. During rapid growth phases such as fruit enlargement, grain filling, and fiber elongation, the SUS-driven production of UDP-Glc fuels the synthesis and deposition of cell wall polysaccharides, thus ensuring organ expansion and morphogenesis (Figure 2) [30,31,32,33]. Classical genetic studies in maize further support the importance of SUS during endosperm filling. In developing maize endosperm, the two major sucrose synthase isozymes encoded by *Sh1* and *Sus1* make distinct but complementary contributions to kernel development: the Sh1 product is more closely associated with the maintenance of endosperm cell wall integrity, whereas the Sus1 product is particularly important for starch biosynthesis and accumulation [34]. Accordingly, disruption of these sucrose synthase isozymes impairs endosperm development and reduces starch deposition, providing classical genetic evidence that SUS activity is required for normal starch accumulation during maize seed filling [34]. In cotton, this role is supported by classical functional evidence showing that the suppression of sucrose synthase expression represses fiber cell initiation and elongation and impairs seed development, demonstrating that SUS is required for normal cotton fiber development [35].

Under stress conditions, SUS also functions as an important metabolic buffer and regulatory node [6,21]. In the presence of adverse factors such as drought, low temperature, hypoxia, and salinity, SUS modulates the levels of sucrose and various soluble sugars to maintain cellular osmotic adjustment and carbon metabolic homeostasis, thereby contributing to enhanced stress tolerance and metabolic stability and improving plant adaptation to extreme environments [36,37,38].

At the metabolic level, SUS is involved not only in the biosynthesis of primary metabolites, including sucrose, hexoses, UDP-Glc and cellulose, but also in bridging primary and secondary metabolism [16,22]. For example, in medicinal plants such as licorice (*Glycyrrhiza* spp.), SUS-mediated control of UDP-sugar supply influences the biosynthesis of glycyrrhizin and other specialized metabolites, providing an important metabolic basis for disease and pest resistance as well as ecological adaptation [39,40,41,42].

The functions of SUS further extend into signaling regulation. Several phytohormones, including gibberellins (GAs), abscisic acid (ABA), brassinosteroids (BRs), and ethylene, regulate carbon partitioning, energy utilization, and developmental processes by modulating the transcription of SUS genes [15,21,43,44]. In particular, ethylene-responsive transcription factors (ERFs) constitute an important regulatory layer in this process. For example, the cassava ethylene-responsive factor MeERF72 negatively regulates the expression of the cassava sucrose synthase 1 gene, providing direct evidence that ethylene signaling can reshape SUS-mediated carbon allocation through ERF-family transcription factors [44]. In addition, SUS expression is also controlled by key transcription factors such as Opaque2 and CAMTA proteins, creating multi-layered crosstalk between metabolic and signaling pathways and positioning SUS as a core regulatory node that links metabolic homeostasis, developmental control, and stress responses [33,45,46,47,48].

SUS also plays a non-negligible role in determining crop quality [21]. By affecting the accumulation of sucrose and its derivative metabolites, SUS contributes to fruit sweetness and flavor, grain filling efficiency in cereals, and cell wall deposition in fiber crops [28,49,50,51]. The impact of SUS on these quality-related traits makes the SUS gene family an attractive target for molecular breeding strategies aimed at the simultaneous improvement of yield and quality [48,52].

Taken together, the SUS gene family constitutes an integrated functional network spanning assimilate transport, carbon partitioning, energy metabolism, stress responses, primary and secondary metabolism, hormone signaling, and crop quality regulation [11,21,52]. SUS is thus not only a key catalytic enzyme in plant carbon metabolism but also an important integrative component linking carbon metabolism with plant growth, development, and environmental responses. As the molecular mechanisms underlying SUS gene function are further elucidated, targeted manipulation of SUS gene expression or SUS activity is expected to provide new conceptual frameworks and technical strategies for improving crop stress tolerance, yield, and quality [3,21,37,52]. The above functions provide a general physiological framework for understanding SUS activity, whereas the underlying molecular regulatory mechanisms are discussed in Section 5.

## 3. Organ-Specific Expression and Subcellular Localization of SUS Genes

### 3.1. Organ-Specific Expression Patterns of SUS Genes

**Figure 3:**
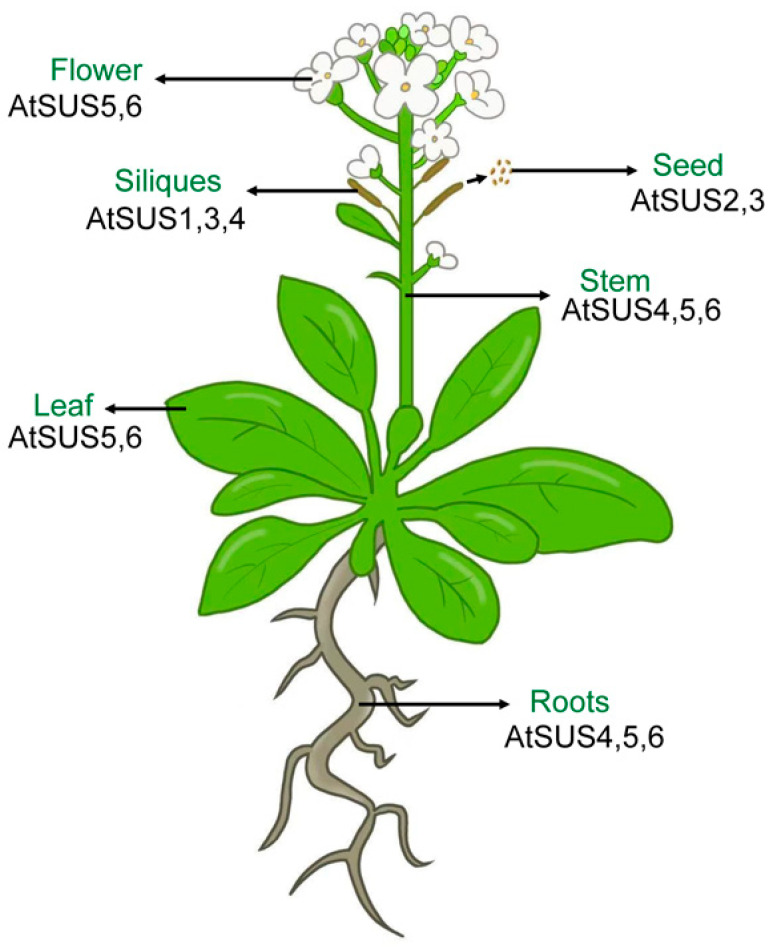
Schematic representation of the relative expression levels of the SUS gene family in major organs of *Arabidopsis thaliana.* This schematic summarizes the organ-specific expression profiles of the six *AtSUS* isoforms in major vegetative and reproductive organs of *Arabidopsis thaliana*, based on qRT-PCR data normalized to EF1A4a and expressed as %EF. The figure highlights the marked functional differentiation of individual *AtSUS* members across roots, stems, flowers, siliques, and developing seeds.

The SUS gene family in *Arabidopsis thaliana* exhibits a pronounced organ-specific expression pattern, in which distinct *AtSUS* members form a spatially differentiated expression network between vegetative and reproductive organs (Figure 3). Based on qRT-PCR analysis normalized to *EF1A4a*, with transcript abundance expressed as a percentage of *EF1A4a* expression (%EF) [53], the six isoforms displayed clear expression gradients across major organs and showed marked developmental stage-dependent regulation.

*AtSUS1* maintains relatively low transcript levels across the examined organs and developmental stages, with only a slight increase in the silique wall during development.

*AtSUS2* is the most seed-specific member during maturation. It is barely detectable at 6 days after flowering (DAF) (<0.1% EF), but its expression rises sharply to about 80% EF at 12 DAF, coinciding with the rapid accumulation of storage reserves, and then drops markedly by 15 DAF and is nearly absent at 18 DAF. This “burst-like” expression pattern closely parallels the period of oil and protein deposition, indicating that *AtSUS2* plays a central role in supplying carbon skeletons and supporting storage metabolism during seed filling.

*AtSUS3* is predominantly expressed in late developmental tissues, including the silique wall at 15–18 DAF and desiccated mature seeds. Its transcript level increases markedly during silique wall yellowing. During seed germination, *AtSUS3* expression is about 20% EF at 0 h and declines to approximately 1.6% EF by 24 h, thereafter remaining low and stable. This pattern suggests that *AtSUS3* may be associated with late seed maturation, energy management, and the transition from seed maturation to germination.

*AtSUS4* is mainly expressed in vegetative organs, with the highest levels in roots (approximately 11% EF) and stems (approximately 3.4% EF), whereas its expression in flowers, leaves, and siliques is relatively low. This expression profile indicates that *AtSUS4* preferentially supports sucrose utilization, carbon flux, and energy supply in vegetative tissues.

*AtSUS5* and *AtSUS6* form a distinct clade within the *SUS* family. At the organ level, both genes are detectable across the tissues examined and show relatively broad expression patterns, with only a slight decrease in the silique wall at late developmental stages [53]. However, this broad organ-level detectability should not be interpreted as a lack of cellular specialization, because tissue-print and localization analyses have shown that *AtSUS5* and *AtSUS6* are closely associated with phloem tissues, particularly sieve elements [54]. The two proteins are of relatively high molecular mass and share 69.5% amino acid identity, suggesting partially overlapping but specialized roles in phloem carbon metabolism and related processes.

Overall, the SUS gene family in *Arabidopsis* operates through mutually independent yet complementary expression patterns. Different *AtSUS* members undertake distinct metabolic roles in vegetative organs, late developmental tissues, and maturing seeds, providing a finely tuned transcriptional basis for efficient carbon flux regulation in response to diverse physiological demands [21,52,53,55].

Recent transcriptomic resources now provide a broader and higher-resolution context for interpreting these classical *Arabidopsis* expression patterns [53,56]. In *Arabidopsis*, platforms such as the Bio-Analytic Resource (BAR) ePlant/eFP browsers integrate tissue-level RNA sequencing (RNA-Seq) compendia, and more recently, root single-cell RNA-Seq data, allowing *AtSUS* expression to be revisited beyond the original 2004 qRT-PCR framework [56]. More generally, large-scale plant expression resources such as PlantExp now enable cross-species comparison of *SUS* transcription across diverse tissues, developmental stages, and experimental conditions [57]. Consistent with the *Arabidopsis* evidence for strong spatiotemporal diversification, RNA-Seq-based studies in other species have also revealed marked spatiotemporal diversification of SUS family members. For example, in pomegranate, *PgSUS1*, *PgSUS3*, and *PgSUS4* are highly expressed in sink organs and are particularly enriched during seed-coat development [29], whereas in potato, *StSUS* genes show differentiated expression across developmental tissues, with *StSUSIc* displaying the highest overall expression in the available datasets [9]. In maize, recent spatial transcriptomics during kernel filling further identified *SH1* among marker genes associated with starch-biosynthesis-related regions, supporting the view that SUS expression is not only organ-specific but can also be resolved at the sub-organ and tissue-domain levels [58]. Together, these newer datasets indicate that the classical *Arabidopsis* observations remain informative, but they should now be interpreted within a broader multi-species and increasingly high-resolution transcriptomic framework [53,56,57,58].

### 3.2. Subcellular Localization and Functional Specialization

**Figure 4:**
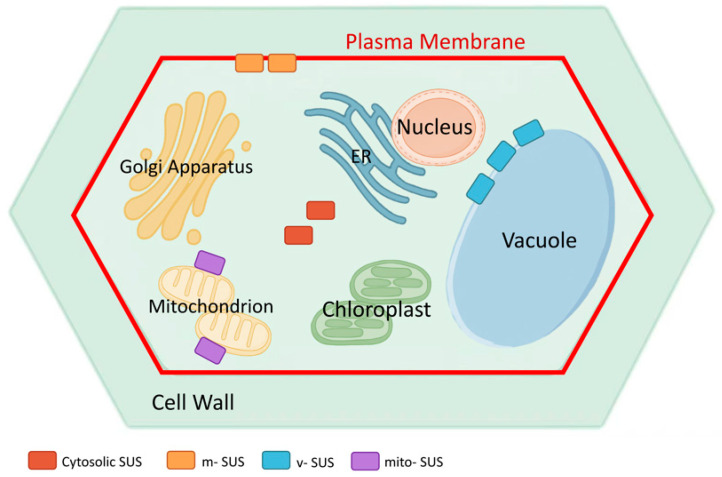
Schematic representation of subcellular localization patterns of sucrose synthase in plant cells. Sucrose synthase occurs as a soluble cytosolic enzyme, cytosolic SUS (red), and in several membrane-associated forms: plasma membrane-bound SUS, m-SUS (orange); tonoplast-localized SUS in sugar-storing tissues, v-SUS (blue); and mitochondrial outer membrane-associated SUS that can be recruited under stress conditions, mito-SUS (purple). The plasma membrane is indicated by a solid red line. This schematic summarizes reported SUS localizations within a generic plant cell, as inferred from published experimental approaches including fluorescent protein fusion imaging, membrane fractionation, immunogold labeling, subcellular fractionation, and co-localization analyses.

Sucrose synthase displays diverse subcellular localization patterns in plant cells (Figure 4), and this spatial distribution is closely linked to its roles in carbon flux regulation, structural carbon biosynthesis, and energy metabolism [21,52]. In most plant species, the predominant form of SUS is the soluble cytosolic enzyme (cytosolic SUS), which is often described as a tetrameric enzyme and catalyzes the reversible interconversion between sucrose + UDP and UDP-Glc + fructose, although available evidence indicates that SUS oligomerization can vary among isoforms and species [10,21,24]. The intracellular localization patterns summarized here were inferred from published experimental evidence, including fluorescent protein fusion imaging, membrane fractionation, immunogold labeling, subcellular fractionation, and co-localization analyses.

In the model plant *Arabidopsis thaliana*, systematic imaging of yellow fluorescent protein (YFP) fusion proteins has revealed that all six SUS isoforms (*AtSUS1*–*AtSUS6*) are localized to the cytosol, with no detectable signals at the plasma membrane, tonoplast, or mitochondrial membranes [26,59]. *AtSUS1* and *AtSUS4* show diffuse cytosolic distribution in companion cells, whereas *AtSUS5* and *AtSUS6* display peripheral cytosolic signals in sieve elements, consistent with the phloem sieve element-specific distribution reported by Barratt et al. [26,53,54]. This phloem-associated localization is notable because earlier genetic evidence suggested that phloem-localized SUS may contribute to callose synthesis at sieve plates. In particular, the *sus5 sus6* double mutant showed reduced callose accumulation in sieve plates [54], supporting the hypothesis that these phloem-expressed SUS isoforms help provide UDP-Glc for callose deposition in the phloem [54]. Consistent with this interpretation, CALLOSE SYNTHASE 7 (CALS7/GSL7) was later identified as a phloem-specific callose synthase required for callose deposition in developing sieve elements and in mature phloem [60]. *AtSUS2* and *AtSUS3* appear as punctate structures in endosperm and embryo cells. Notably, none of these isoforms co-localize with the cellulose synthase complex (CSC), nor are they significantly enriched at sites of active cell wall deposition, indicating that *Arabidopsis* SUS mainly contributes to sucrose loading/unloading and energy provision rather than directly supplying UDP-Glc for cellulose biosynthesis [11,27]. This interpretation is further strengthened by the work of Wang et al., who generated *Arabidopsis* mutants lacking four or all six SUS genes and showed that even the sextuple mutant displayed no obvious growth phenotype, no vascular cell wall defects, and no reduction in cellulose content under standard growth conditions [27]. These findings suggest that, at least in *Arabidopsis*, SUS activity is dispensable for normal growth and development under non-stress conditions, and that alternative sucrose-cleavage pathways or broader metabolic redundancy may compensate for the loss of SUS function [27]. In contrast, the phenotypic consequences of SUS perturbation are much stronger in some cereal systems. In maize, loss of *SH1* markedly reduces endosperm sucrose synthase activity, disrupts carbon partitioning, and causes severe kernel defects, while additional disruption of *SUS1* and *SUS2* further aggravates endosperm filling phenotypes [45,61]. In rice, perturbation of SUS function also produces clear physiological consequences, for example, reduced drought tolerance in *ossus1* mutants, indicating that the developmental and physiological requirement for SUS is species- and context-dependent rather than universal across plants [37]. Although *AtSUS1* and *AtSUS4* possess the capacity for reversible plasma membrane association mediated by phosphorylation of a conserved N-terminal serine, they still exist predominantly as cytosolic enzymes under steady-state conditions [62,63].

In contrast to *Arabidopsis*, SUS exhibits more complex membrane-related localization patterns in other plant species. The earliest evidence for plasma membrane association came from cotton fiber cells. Membrane fractionation and immunogold labeling studies showed that approximately half of the SUS protein is anchored to the cytosolic face of the plasma membrane, forming band-like distributions along the axis of fiber elongation in close proximity to the sites of cellulose microfibril deposition [64,65]. Subsequent studies demonstrated spatial overlap between SUS signals and CSC-rich regions, providing molecular evidence for the membrane-bound form (m-SUS) and suggesting that m-SUS locally supplies UDP-Glc for cellulose synthesis. This membrane association is linked to the phosphorylation status of the N-terminal serine residue, enabling SUS to shuttle dynamically between the cytosol and plasma membrane and thereby adapt to changes in the rate of cell wall synthesis [47,66,67].

A vacuolar form of SUS (vacuolar SUS, v-SUS) has been identified in sugar-storing fruit tissues. Subcellular fractionation combined with GFP fusion protein tracking showed that grape VvSUS3 is localized to the tonoplast and can interact with the β-subunit of SNF1-related protein kinase 1 (SnRK1). The phosphorylation status of VvSUS3 influences soluble sugar accumulation and osmotic adjustment, indicating that v-SUS plays an important role in sugar storage and cellular osmotic homeostasis [49,50].

Under specific stress conditions, a mitochondrial-associated form (mitochondrial SUS, mito-SUS) can also be observed. In maize pollen, part of the SUS signal is detected at the outer mitochondrial membrane and is markedly enhanced under combined heat and drought stress [38]. Based on current models, mito-SUS is proposed to form functional modules with respiratory enzyme complexes to reinforce sugar catabolism and energy regeneration under stress conditions [37,38].

Taken together, SUS exhibits a wide range of subcellular localization forms across different plant species: all six SUS isoforms in *Arabidopsis* are cytosolic, whereas cotton, tomato, grape, and maize possess additional plasma membrane-associated, tonoplast-associated, and mitochondrial-associated SUS pools, together with more weakly resolved peripheral signals reported in some tissues. These spatially distinct distributions reflect fine functional partitioning of SUS in directing carbon fluxes, maintaining energy balance, and supporting structural carbon biosynthesis, and they highlight the high degree of flexibility in SUS-mediated metabolic regulation in response to environmental changes [11,21,52].

In addition to the major cytosolic and membrane-associated pools described above, some studies have reported further subcellular associations of SUS. These include association with the actin cytoskeleton [68], strong plastid-related localization of *AtSUS2* in developing *Arabidopsis* seeds [69], and Golgi membrane enrichment in certain species such as maize [70]. However, these localization patterns appear to be species-, isoform-, and tissue-dependent, and are less broadly established than the major cytosolic and plasma membrane-associated forms [26,71].

## 4. Phylogenetic Relationships and Evolutionary Patterns of the SUS Gene Family

### 4.1. Gene Family Composition and Cross-Species Distribution of SUS Genes

**Figure 5:**
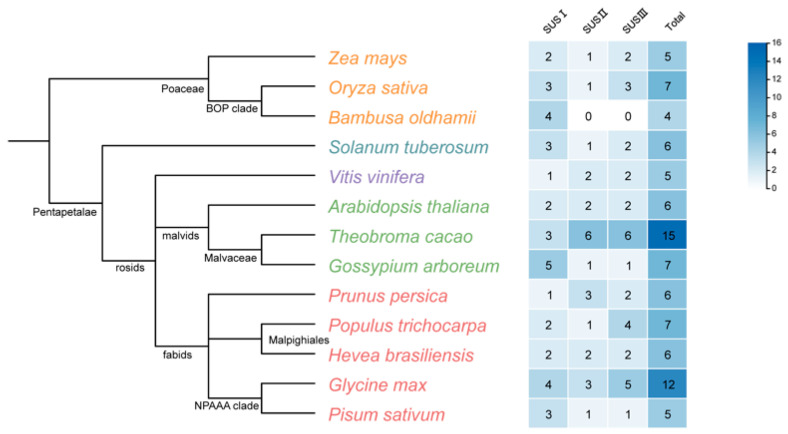
Number and subgroup distribution of SUS gene family members in selected angiosperm species. Figure compares SUS family size and clade composition among 13 selected species, highlighting lineage-specific variation in total copy number and the uneven expansion of the three canonical subgroups, SUS I, SUS II, and SUS III.

To elucidate the diversity and evolutionary patterns of the sucrose synthase gene family, SUS protein sequences from 13 selected species were retrieved from the NCBI database. Statistical analysis (Figure 5) revealed pronounced variation in SUS copy number among species, with total gene numbers ranging from 4 to 15, and a clear lineage-specific distribution pattern [9].

Among the dicotyledonous species, legumes and malvaceous species generally harbor larger SUS families. For example, soybean (*Glycine max*) contains 12 SUS members, and cacao (*Theobroma cacao*) possesses as many as 15 SUS genes, representing the largest family size in our dataset [72]. Such striking expansions are typically associated with segmental duplication and/or whole-genome duplication (WGD) events [21,29]. In contrast, monocotyledonous plants such as rice (*Oryza sativa*) and maize (*Zea mays*) carry 7 and 5 SUS genes, respectively, and overall show relatively stable family sizes, reflecting a higher degree of conservation of the SUS family within monocot lineages [45,73].

With respect to clade composition, the three canonical SUS subgroups—SUS I, SUS II and SUS III—exhibited uneven expansion across species. For instance, SUS II has undergone marked expansion in cacao. In contrast, in our current dataset, only SUS I members were recovered from *Bambusa oldhamii*. However, the apparent absence of SUS II and SUS III in bamboo should be interpreted cautiously rather than as definitive evidence of true lineage loss. In *Bambusa oldhamii*, at least four sucrose synthase cDNAs have previously been cloned [74]. Therefore, this pattern may reflect incomplete genome assemblies or annotations, transcriptome/genome sampling limitations, or stringent sequence-retrieval criteria, in addition to the possibility of lineage-specific gene loss. More exhaustive searches based on reciprocal BLAST/tBLASTn, conserved-domain validation, and updated genome annotations will be required before true subgroup absence can be inferred with confidence [53,72].

### 4.2. Phylogenetic Relationships and Evolutionary Divergence

**Figure 6:**
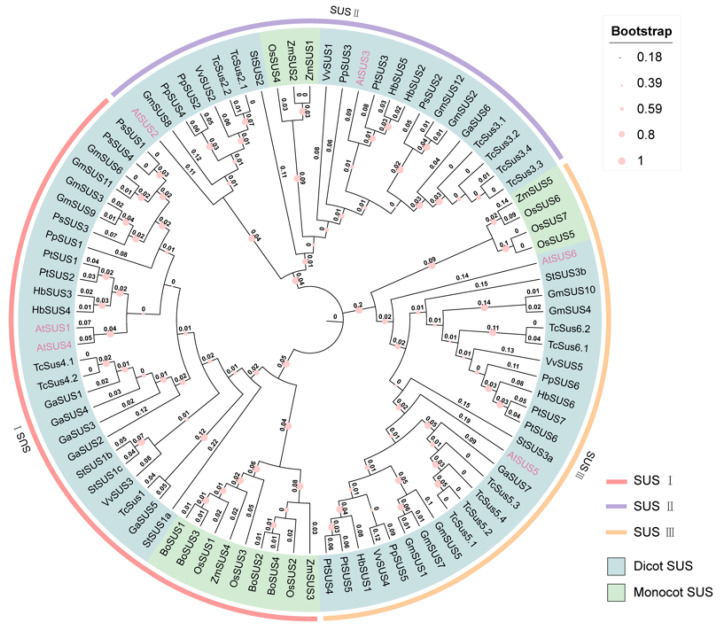
Neighbor-joining phylogenetic tree of the *SUS* gene family in selected plant species. The NJ tree was constructed in MEGA 11 using full-length SUS protein sequences from 13 species, with the Poisson correction model and 1000 bootstrap replicates. The tree resolves SUS members into three major clades (SUS I, SUS II, and SUS III). Branch length values are shown on the tree to facilitate the interpretation of evolutionary divergence among SUS members.

Based on full-length SUS protein sequences from 13 plant species, a neighbor-joining (NJ) phylogenetic tree was constructed using MEGA 11 (Figure 6). The resulting topology clearly resolved the SUS family into three major clades: SUS I, SUS II, and SUS III [6,53].

The SUS I clade contains both monocot and dicot sequences, which form two well-supported lineage-specific subgroups, indicating clear divergence between monocotyledonous and dicotyledonous plants. Branch lengths within SUS I vary considerably, indicating that gene duplication and subsequent expansion have occurred during the evolution of this clade [29,72]. SUS II and SUS III also include both monocot and dicot sequences [6].

In *Arabidopsis thaliana*, the six SUS genes are distributed across the three clades: *AtSUS1* and *AtSUS4* cluster within SUS I; *AtSUS2* and *AtSUS3* belong to SUS II; and *AtSUS5* and *AtSUS6* fall into SUS III. These clade assignments correspond well to their distinct expression patterns, subcellular localizations, and physiological roles [53,59]. SUS I genes are predominantly expressed in vegetative tissues such as roots and stems, where they participate in carbon flux and energy metabolism. SUS II genes are strongly upregulated during seed development and storage reserve accumulation, serving as key providers of UDP-Glc and carbon skeletons for storage metabolism. SUS III genes are expressed in multiple tissues, but are frequently associated with cell wall metabolism, signaling pathways, and stress responses, suggesting that they act as important nodes integrating structural carbon biosynthesis with environmental adaptation [11,52].

Overall, phylogenetic analysis indicates that although the SUS family is highly conserved at the domain architecture level, lineage-specific duplication and divergence events have generated functionally differentiated subgroups in different species and tissues. This evolutionary diversification provides a multilayered basis for the fine-tuned regulation of plant carbon metabolism [11,21].

### 4.3. Cis-Regulatory Divergence in the Promoter Regions of Arabidopsis SUS Genes

**Figure 7:**
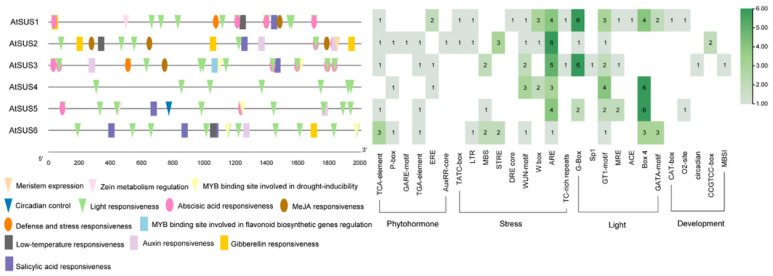
Cis-regulatory divergence in the promoter regions of *AtSUS1*–*AtSUS6* in *Arabidopsis thaliana*. (**Left**), positional distribution of predicted cis-elements within the 2-kb upstream promoter regions of the six *AtSUS* genes, as identified using the PlantCARE database. (**Right**), summary heatmap showing the relative abundance of representative cis-elements grouped into phytohormone-responsive, stress-responsive, light-responsive, and development-related categories. Blank cells indicate the absence of the corresponding cis-element.

In addition to divergence at the coding-sequence and phylogenetic levels, regulatory diversification of the *Arabidopsis thaliana* SUS family may also be reflected in promoter architecture. To further examine this aspect, the 2-kb upstream promoter regions of *AtSUS1*–*AtSUS6* were comparatively analyzed for predicted cis-regulatory elements using the PlantCARE database (Figure 7). The results revealed both shared and gene-specific distributions of cis-elements associated with phytohormone responsiveness, stress signaling, light responsiveness, and developmental regulation. Several cis-elements were widely represented across multiple *AtSUS* promoters, whereas others showed uneven abundance and positional variation among individual genes. These differences suggest that cis-regulatory divergence may have contributed to the distinct expression patterns and functional specialization of *AtSUS* members during evolution. Together with the phylogenetic divergence, these promoter-level differences suggest that the functional diversification of the SUS gene family in *Arabidopsis* may result from the combined effects of coding-sequence evolution and cis-regulatory divergence.

## 5. Multi-Level Regulatory Mechanisms of *SUS* Genes

As a key component of plant carbon metabolism, sucrose synthase is embedded in a regulatory framework that spans multiple molecular layers [18]. Rather than being controlled solely at the transcriptional level, the functional state of SUS is jointly shaped by the transcriptional, post-transcriptional, post-translational, protein-interaction, and subcellular organization levels, which together determine its abundance, localization, stability, and catalytic behavior [11,52,67]. Upstream sugar signals, cellular energy status, and hormone pathways define the transcriptional potential of SUS genes; miRNA-mediated control of mRNA stability adds an additional layer of precision to SUS expression [1,45,46,75]. Post-translational modifications such as phosphorylation and ubiquitination allow SUS to switch within short time scales between cytosolic and membrane-associated forms and to fine-tune its stability and catalytic properties [37,62,66,76]. Interactions with cytoskeletal elements and other metabolic enzymes provide spatial anchoring and metabolic channeling, thereby influencing the molecular context in which SUS functions [13,55,67]. Together, these regulatory layers shape how SUS is deployed and regulated under specific developmental and environmental conditions [11].

### 5.1. Transcriptional Regulation of SUS Genes

**Figure 8:**
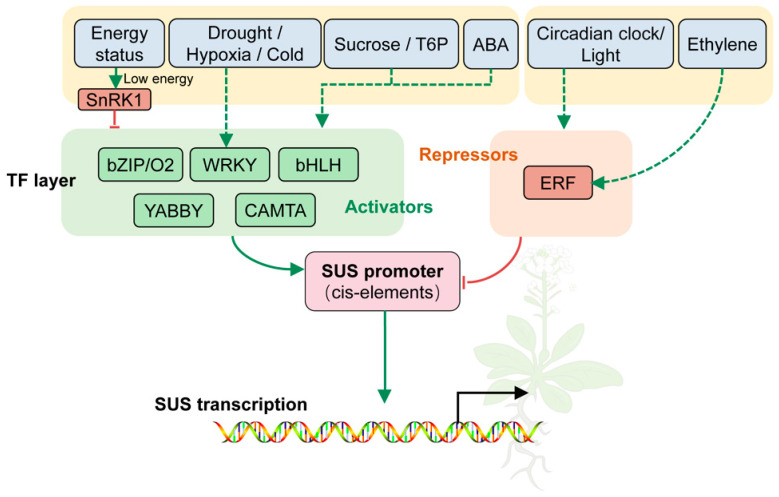
Multi-level regulatory network controlling SUS transcription in plants. This schematic summarizes the major upstream inputs and regulatory layers that modulate SUS gene expression, including sugar and energy signals, hormone pathways, environmental cues, transcription factors, and post-transcriptional regulators. Together, these components form an integrated regulatory framework that dynamically adjusts SUS transcription during carbon partitioning, sink establishment, development, and stress responses.

The expression of sucrose synthase genes is subject to finely tuned control by multiple signaling inputs and molecular mechanisms, forming a complex regulatory network that integrates metabolic status, hormonal cues, and environmental information (Figure 8) [21,52]. This network can be conceptualized as a multilayered regulatory framework in which upstream signals shape the transcriptional competence of SUS loci, transcription factors modulate promoter activity, and additional regulatory processes further refine SUS transcript abundance and temporal responsiveness, thereby enabling dynamic adjustment of SUS expression during carbon partitioning, sink establishment, and stress responses [11,52]. However, these pathways should not be interpreted as having been universally demonstrated for all three SUS clades (SUS I–III) or for all isoforms within a given species. At present, most mechanistic evidence derives from selected SUS members in particular species, tissues, and developmental contexts, whereas for many other isoforms, the proposed regulatory relationships remain inferred from expression patterns, promoter composition, or broader signaling frameworks.

At the level of upstream signals, sugar signaling, cellular energy status, hormone pathways, and environmental stimuli collectively determine the expression potential of SUS genes. Sucrose and its derived signals, such as trehalose-6-phosphate (T6P), modulate SUS transcription through energy-sensing systems [2,75]. In maize endosperm, sucrose-associated SnRK1a1 signaling modulates Opaque2 activity and thereby influences the transcription of *Sus1* and *Sus2* [33,45]. Hormones including abscisic acid (ABA) and ethylene dynamically reorient carbon fluxes during grain filling, fruit development, and stress, and reshape the spatiotemporal expression patterns of SUS genes [3,15,43,44]. Environmental cues such as low temperature, drought, and hypoxia also induce stress-responsive transcriptional programs that reprogram SUS expression under adverse conditions to match cellular energy and carbon demands [36,38].

At the core transcription-factor layer, available evidence supports the regulation of specific SUS members rather than a universal mechanism shared by all SUS isoforms. For example, bZIP/Opaque2-type regulation has been demonstrated most clearly in maize, where Opaque2 directly activates *Sus1* and *Sus2* during endosperm filling, thereby promoting UDP-Glc supply and storage metabolism [33,45]. Ethylene-related repression is supported by the cassava *MeERF72*–*MeSUS1* module, in which *MeERF72* negatively regulates the expression of *MeSUS1* [40]. More broadly, recent reviews indicate that ERF-family repressors can contribute to stress-induced carbon reallocation and growth–stress trade-offs, thereby helping redirect carbon fluxes under adverse conditions [77]. A further isoform-resolved example is provided by plum, where bHLH-mediated regulation has been reported for *PsSUS4* in association with fruit sugar accumulation [51]. In addition, CAMTA proteins may participate in SUS transcriptional regulation together with chromatin-modifying factors. In wheat, CAMTA2 has been shown to cooperate with GCN5 in activating *Sus2*, supporting a link between Ca^2+^-responsive transcriptional control and chromatin-mediated regulation of SUS expression [48]. More recently, a combined YABBY–WRKY regulatory module has been reported in sweet sorghum, where SbYABBY8 and SbWRKY23 transactivate *SbSuSy1* and promote sucrose accumulation in stalk tissues, further supporting the view that the transcriptional regulation of SUS can also involve the coordinated action of multiple transcription factors in a species- and isoform-specific manner [78].

Collectively, the transcriptional control of SUS genes is characterized by multi-signal integration, coordinated action of diverse transcription factors, and extensive cross-talk between regulatory layers. This network effectively couples metabolic status, environmental fluctuations and developmental programs, allowing plants to fine-tune sucrose-cleaving capacity and carbon partitioning strategies and thereby maintain an appropriate balance between growth, storage, and stress tolerance [11,52]. At the same time, current evidence indicates that many of these regulatory relationships are isoform-specific rather than universally established across the entire SUS family.

### 5.2. Post-Transcriptional Regulation of SUS Genes

**Figure 9:**
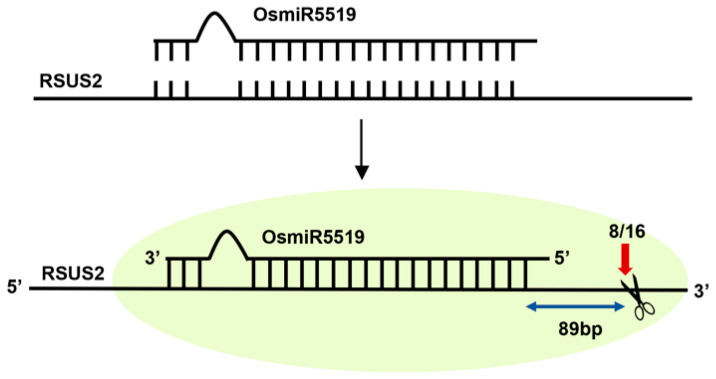
Schematic model of the atypical OsmiR5519-mediated cleavage of *RSUS2* (*OsSUS2*). OsmiR5519 binds to the complementary region within the *RSUS2* transcript but does not cleave at the canonical miRNA cleavage site. Instead, it cleaves 89 bp downstream of this position, as confirmed by 3′-RACE analysis (8 out of 16 clones).

Within the regulatory network of the sucrose synthase gene family, current evidence at the post-transcriptional level has so far mainly focused on miRNA-mediated control [21]. In rice, OsmiR5519 has been shown to directly recognize and cleave the *RSUS2* (*OsSUS2*) transcript. This interaction was confirmed by RNA ligase-mediated rapid amplification of cDNA ends (RLM-RACE) and dual-luciferase reporter assays (Figure 9). High levels of OsmiR5519 markedly reduce *RSUS2* mRNA abundance, concomitant with a decrease in soluble sugar content and impaired grain filling, whereas suppression of this miRNA restores *RSUS2* expression and improves grain development. This miRNA–SUS regulatory module provides direct molecular evidence that members of the SUS family are subject to fine post-transcriptional control, and it underscores their importance in carbon reallocation and sink formation [1,52].

### 5.3. Phosphorylation-Mediated Regulation of SUS Genes

Sucrose synthase is a key hub in the plant carbon metabolic network, and its enzymatic activity, subcellular localization, and protein stability are all subject to fine regulation by phosphorylation at multiple levels [21,47,63,66,79]. The major phosphorylation sites of SUS are clustered in the N-terminal intrinsically disordered region, particularly in a highly conserved Ser-11–Ser-15 stretch, and in some species, around Ser-170. These residues are broadly conserved in land plants and have corresponding motifs in algal SUS homologs [6]. Accumulating evidence indicates that the phosphorylation of N-terminal Ser residues not only alters local SUS conformation but also introduces a stable negative charge that affects the catalytic microenvironment, thereby modulating kinetic properties in the sucrose-cleaving direction and the efficiency of UDP-Glc production. In vitro kinetic analyses consistently show that phosphorylation of the N-terminal Ser sites enhances SUS activity in the sucrose-cleaving direction and increases its capacity to supply UDP-Glc. In functional mutagenesis studies, phosphomimetic substitution (S→D), which stabilizes a phosphorylation-like state, generally leads to a marked increase in SUS catalytic efficiency, whereas non-phosphorylatable substitution (S→A) is associated with reduced activity [47,62,66]. Together, these findings support a central role of N-terminal phosphorylation in controlling SUS activity and metabolic flux direction [21]. However, although these observations indicate that phosphorylation is an important regulatory mechanism of SUS, current direct evidence is still limited to selected isoforms and species, and thus should not be interpreted as universally demonstrated for all SUS members or all three SUS clades.

Importantly, the phosphorylation status of SUS also determines its subcellular distribution [37,66,67]. Dephosphorylated SUS preferentially associates with the plasma membrane, where it contributes to cell wall polysaccharide synthesis, whereas phosphorylated SUS is predominantly cytosolic and favors carbon flux toward starch biosynthesis, respiration, and other energy-producing pathways [63,64,65]. Thus, SUS phosphorylation not only regulates its enzymatic properties but also reshapes the overall direction of carbon flux by influencing its compartmentalization [18,66].

**Figure 10:**
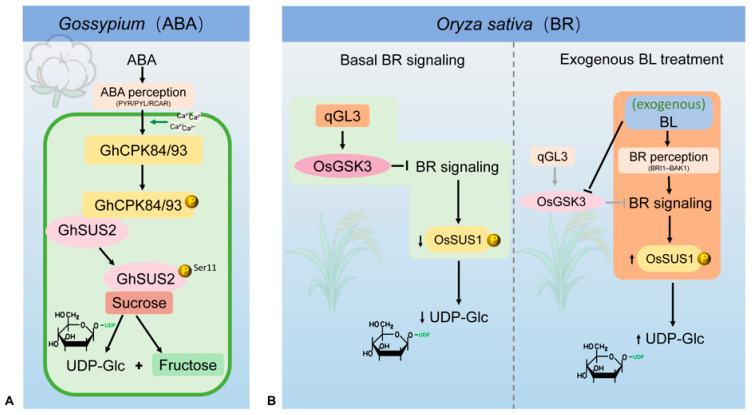
Simplified schematic representation of hormone-mediated SUS phosphorylation in cotton and rice. This figure illustrates two representative signaling modules by which ABA and BR signaling regulate SUS phosphorylation in *Gossypium* and *Oryza sativa*. (**A**) ABA-related module in *Gossypium*. ABA perception, represented here at the receptor level, is associated with a rise in cytosolic Ca^2+^, activation of the Ca^2+^-dependent protein kinases GhCPK84 and GhCPK93, and phosphorylation of Gh*SUS2* at Ser11, thereby stimulating sucrose cleavage to UDP-Glc and fructose [47]. (**B**) BR-related module in *Oryza sativa*. BR perception, represented here at the receptor level, activates BR signaling, relieves OsGSK3-mediated repression, and promotes *OsSUS1* accumulation and phosphorylation, leading to elevated UDP-Glc supply [80,81,82,83].

In planta, SUS phosphorylation is modulated by several hormone and signaling pathways, among which abscisic acid (ABA) and brassinosteroids (BRs) are currently the best characterized [15,21,37]. In cotton, ABA treatment rapidly elevates cytosolic Ca^2+^ levels, thereby activating the Ca^2+^-dependent protein kinases GhCPK84 and GhCPK93 [47]. These kinases directly phosphorylate *GhSUS2* at Ser-11, promoting SUS dissociation from the plasma membrane into the cytosol and markedly enhancing its sucrose-cleaving activity and the production of UDP-Glc and fructose. This supports a representative signaling module linking ABA signaling to SUS phosphorylation and carbon reallocation (Figure 10A) [47], and identifies SUS as an important effector through which ABA drives carbon reallocation.

In rice, phosphorylation of SUS is controlled primarily through hormone signaling cascades rather than direct kinase action (Figure 10B). The qGL3 locus (OsPPKL1) encodes a negative regulator in the BR pathway that stabilizes OsGSK3 and thereby maintains strong repression of BR signaling, resulting in reduced *OsSUS1* expression, a lower phosphorylation level of *OsSUS1* protein and insufficient UDP-Glc supply [80,81]. Upon exogenous application of brassinolide (BL), BL directly inhibits OsGSK3 activity, relieving its repression of BR signaling and allowing BR signaling output to increase [80,81,82]. Consequently, the *OsSUS1* transcript and protein levels rise substantially [1]. *OsSUS1* variants with different phosphorylation capacities (SS, phosphorylatable; DD, phosphomimetic; AA, non-phosphorylatable) show distinct accumulation patterns in response to BL: SS and DD accumulate most strongly, whereas AA shows the weakest increase, indicating that the phosphorylation state markedly enhances *OsSUS1* stability and its responsiveness to BR signaling [73]. The accompanying increase in UDP-Glc promotes BR-dependent traits such as improved grain filling, increased thousand-grain weight, and changes in leaf angle [1,73]. Together, these observations delineate a regulatory chain in rice in which “BL → inhibition of OsGSK3 → enhanced BR signaling → increased stability/expression of *OsSUS1* → elevated UDP-Glc”.

In summary, SUS phosphorylation represents one of the core mechanisms by which plants integrate hormonal signals with carbon metabolism [6]. By directly influencing SUS activity, conformation, and membrane association, and by modulating SUS abundance and stability through ABA, BR, and Ca^2+^–kinase networks, phosphorylation precisely controls the supply of UDP-Glc and the direction of carbon flux. Overall, phosphorylation-dependent regulation appears to be a functionally important mode of SUS control in plants; however, the mechanistically resolved examples currently involve only a limited number of isoforms and species, and therefore this mechanism should not yet be regarded as universally established across the entire SUS family [11,21].

In addition to phosphorylation, SUS turnover may also be associated with the ubiquitin–26S proteasome pathway [62,79]. However, direct evidence for the ubiquitination-mediated regulation of SUS remains very limited at present [21]. Most current interpretations are based on indirect observations, including the relationship between phosphorylation-dependent SUS destabilization and proteasome activity, as well as broader ubiquitinome studies showing that plant metabolic enzymes can undergo dynamic ubiquitination during metabolic reprogramming [76]. For example, phosphorylation at Ser170 has been associated with reduced SUS stability, suggesting that specific phosphorylated residues may function as potential phosphodegron-like signals linking short-term activity regulation to longer-term protein turnover [62,76,79]. Nevertheless, direct identification of ubiquitinated SUS residues, the responsible E3 ligases, and the mechanistic contribution of ubiquitination to SUS abundance or function is still lacking. Therefore, the role of ubiquitination in SUS regulation should be interpreted cautiously and regarded as a promising but as yet insufficiently validated research direction.

### 5.4. Protein Interactions of SUS

**Figure 11:**
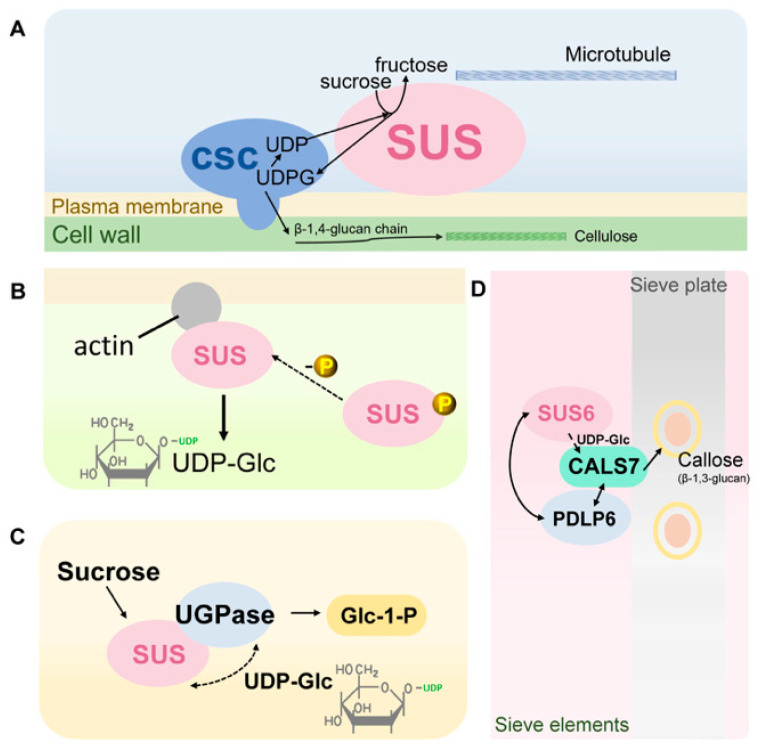
Protein interaction networks of sucrose synthase and their roles in structural localization and carbon flux coupling. (**A**) Model for membrane-associated SUS at the cortical side of the plasma membrane. SUS co-localizes with the CSC and cortical microtubules at the plasma membrane–cell wall interface, where sucrose cleavage supplies UDP-Glc for CSC-driven elongation of β-1,4-glucan chains and cellulose microfibril deposition. (**B**) Phosphorylation-dependent partitioning of SUS between membrane-associated and soluble pools. Dephosphorylated SUS interacts with actin-rich cortical membrane skeleton structures (grey) to form a membrane-bound pool that enhances local sucrose cleavage and UDP-Glc production, whereas N-terminally phosphorylated SUS is depicted as a predominantly soluble cytosolic form. (**C**) Metabolic coupling between SUS and UGPase. Cytosolic SUS generates UDP-Glc from sucrose, and UGPase converts UDP-Glc to Glc-1-P. Dashed lines indicate a hypothetical substrate channeling model within a SUS–UGPase “metabolon” that may support efficient carbon allocation to cell wall precursor synthesis, starch biosynthesis, and other high-flux pathways. This model is inferred based on expression correlation and substrate flow direction and awaits validation by in vitro interaction assays. (**D**) Proposed phloem callose-synthesis module. In the vasculature, phloem-associated SUS6 may contribute local UDP-Glc supply for CALS7-mediated callose deposition at sieve plates. PDLP6 physically and genetically interacts with SUS6, and CALS7 interacts with both SUS6 and PDLP6, supporting a regulatory module that links sucrose cleavage with localized callose accumulation in specialized phloem-associated contexts. Solid lines indicate experimentally supported protein interactions, whereas the dashed arrow indicates proposed local UDP-Glc supply rather than proven direct substrate channeling.

Protein interactions constitute another key regulatory layer for sucrose synthase, complementing transcriptional control and phosphorylation [6,52]. By influencing SUS subcellular distribution, higher-order complex assembly, and the coupling of metabolic pathways, this interaction layer shapes the structural context in which SUS operates in carbon partitioning. Thus, the functional state of SUS depends not only on its expression level and post-translational modifications, but also critically on its interaction partners and spatial organization within the cell (Figure 11) [18,21]. Structural studies further indicate that some aspects of SUS interaction behavior may be associated with its domain architecture, particularly the N-terminal region implicated in cellular targeting and the catalytic GT-B fold that supports substrate binding and functional coordination [23].

First, interactions between SUS, the cytoskeleton, and cell wall biosynthetic factors have been documented in several plant species, particularly in non-Arabidopsis systems [13,67,84]. In these contexts, SUS has been reported to co-localize with cortical cytoskeletal elements and components of the cellulose synthase complex (CSC) [13,64,67]. It has been proposed that SUS, CSC, and cytoskeletal proteins may assemble into a functional multiprotein complex anchored at the cell cortex, in close proximity to sites of cellulose microfibril deposition (Figure 11A) [13,64,65]. Such cytoskeleton-associated anchoring allows SUS to provide a locally directed supply of UDP-Glc at cellulose synthesis “hot spots” and is considered one of the major mechanisms by which SUS sustains localized structural carbon fluxes in rapidly elongating tissues and those undergoing secondary wall thickening [18,65,84].

Second, membrane association of SUS is tightly linked to its N-terminal phosphorylation status and interactions with plasma-membrane-associated cytoskeletal and membrane proteins (Figure 11B). Multiple studies indicate that dephosphorylated SUS preferentially exists in a membrane-associated form, likely enriched on the cytosolic face of the plasma membrane via binding to membrane skeleton components or other membrane proteins [63,66]. In contrast, N-terminal phosphorylation promotes the presence of SUS as a soluble oligomer in the cytosol [63,66]. The membrane-associated form is advantageous in high-flux sink tissues such as tubers, fruits, and filling grains, where efficient sucrose cleavage is required to supply substrates for membrane-associated transport processes and cell wall biosynthesis, whereas the soluble cytosolic form favors the channeling of carbon into glycolysis and energy metabolism [12,26,32,55]. Consequently, phosphorylation-driven membrane association/dissociation, together with reversible interactions with membrane skeleton proteins, is regarded as a crucial molecular switch that dynamically interconverts “membrane-bound” and “cytosolic” SUS functional modes [66,67].

In addition, SUS forms functional modules with various metabolic enzymes and has been widely incorporated into the conceptual framework of “metabolons” (Figure 11C) [16,20,22]. In sucrose degradation pathways, UDP-Glc produced by SUS can be further converted to Glc-1-P by UDP-Glc pyrophosphorylase (UGPase) [20,22]. In sink tissues, the expression and activity of SUS and UGPase often show coordinated changes, leading to the hypothesis that these enzymes may be spatially co-localized or connected by short-range substrate channeling. Such arrangements would tightly couple the production and utilization of UDP-Glc in both time and space, thereby markedly improving substrate transfer efficiency [20,85]. Some studies have further proposed that SUS might participate in larger metabolic networks together with additional key enzymes involved in the synthesis of cell wall precursors or starch, enhancing the capacity to distribute carbon among different metabolic modules, although direct structural and interaction evidence for such higher-order metabolons is still lacking [11,18].

Finally, in addition to its proposed association with cellulose biosynthesis, SUS has also been implicated in callose production, particularly in the phloem. A long-standing hypothesis is that phloem-localized SUS provides UDP-Glc to callose synthase for sieve-plate callose deposition [54]. This idea gained early support from *Arabidopsis*, in which the phloem-expressed *sus5 sus6* double mutant showed reduced callose accumulation in sieve plates [54]. Subsequent work identified CALLOSE SYNTHASE 7 (CALS7/GSL7) as a phloem-specific callose synthase responsible for callose deposition in developing sieve elements and in mature phloem [60]. More recently, Li et al. demonstrated that PLASMODESMATA-LOCATED PROTEIN 6 (PDLP6) physically and genetically interacts with SUS6, and that CALS7 also interacts with both SUS6 and PDLP6, supporting a vascular regulatory module that links sucrose cleavage with callose accumulation in specialized phloem-associated contexts (Figure 11D) [86]. Together, these findings indicate that SUS-mediated UDP-Glc supply may contribute not only to cellulose-related structural metabolism, but also to callose biosynthesis associated with sieve-plate and plasmodesmal function in the vasculature [54,60,86].

Taken together, SUS protein interactions exhibit pronounced spatial dependence, state dependence, and pathway coupling [11,21]. Cytoskeletal anchoring reinforces its role in supplying localized structural carbon fluxes for cellulose-related cell wall construction; membrane skeleton interactions and phosphorylation-dependent switching enable flexible transitions between “membrane-bound” and “cytosolic” functional modes across environmental conditions and developmental stages; metabolon-like assemblies with other metabolic enzymes may enhance substrate utilization efficiency and metabolic integration; and phloem-associated interaction modules involving SUS6, PDLP6, and CALS7 point to an additional role of SUS in supporting callose deposition in specialized vascular contexts [54,60,86]

## 6. Conclusions and Future Perspectives

Sucrose synthase, encoded by the SUS gene family, plays a central role in carbon partitioning, energy metabolism, and structural carbon biosynthesis in plants. Distinct SUS isoforms exhibit pronounced functional diversification in organ-specific expression, subcellular localization, and regulatory patterns, thereby providing context-dependent carbon supply during source–sink establishment, seed development, rapid growth, and stress responses. In parallel, a multi-level regulatory framework—including transcriptional control, miRNA-mediated post-transcriptional regulation, post-translational modifications such as phosphorylation and putative ubiquitination, and protein–protein interactions—collectively determines the activity state and metabolic functions of SUS. Overall, the SUS family combines conserved structural features with substantial functional plasticity, making SUS an important molecular hub linking metabolic homeostasis, developmental regulation, and environmental adaptation, and a representative model for multi-level regulatory networks and functional diversification in plants. With the continuing development of multi-omics technologies, structural biology, and high-resolution cell imaging, our understanding of SUS physiological functions and regulatory mechanisms will deepen and provide a more solid basis for metabolic improvement of crop yield and quality.

Despite considerable progress in elucidating the roles of SUS genes in carbon metabolism and plant development, the overall functional network of SUS is still far from complete, and several key issues require more systematic investigation. First, although previous sections have summarized emerging evidence that SUS stability can be influenced by phosphorylation-dependent protein turnover and by ubiquitin–26S proteasome pathways, the specific mechanisms of SUS ubiquitination remain largely unexplored. In particular, there is still almost no direct evidence identifying ubiquitin acceptor sites on individual SUS isoforms, defining the associated recognition motifs, or characterizing the upstream E2/E3 ligase components involved. Future studies should therefore combine SUS mutant or transgenic materials with ubiquitinome profiling, immunoprecipitation-based ubiquitination assays, proteasome inhibition experiments, and protein degradation kinetics to determine whether and how ubiquitination contributes to SUS turnover and functional regulation in vivo. Second, SUS gene regulatory networks are not yet understood in an integrated manner. Although sugar and energy signals, hormone pathways, and diverse environmental stresses, together with the multiple transcription factor families, miRNA modules, and chromatin-related regulators discussed above, are known to modulate SUS transcription, a unified framework that links these upstream cues to spatially and temporally resolved changes in SUS expression and downstream metabolic responses is still lacking. To move from this fragmented picture to a coherent, systems-level view of SUS regulation, future studies could exploit cutting-edge approaches such as time-resolved single-cell and spatial transcriptomics, next-generation chromatin profiling (including CUT&Tag/CUT&RUN and single-cell multi-omic ATAC-based assays), spatial epigenomics, and CRISPR-based Perturb-Seq or massively parallel reporter assays. In combination with machine-learning-driven regulatory network inference, these strategies will greatly facilitate the precise identification of key transcription factors and cis-regulatory elements in SUS promoters and the dissection of cross-talk logic among distinct signaling pathways. Third, current functional studies are still biased toward primary metabolism, particularly the roles of SUS in sucrose cleavage and resynthesis, the provision of hexoses and UDP-Glc for cellulose and starch biosynthesis, and the support of respiratory energy production in rapidly growing or storage tissues. In contrast, the contribution of SUS to the interface between primary and secondary metabolism through the modulation of UDP-sugar pools remains insufficiently characterized. Existing evidence that SUS affects the biosynthesis and glycosylation of flavonoids, lignans, triterpenes, and triterpenoid-derived specialized metabolites in crops and medicinal plants is relatively scattered, and allelic variation among species and tissues, the diversity of SUS subcellular localizations, and the complexity of environmentally-induced responses further complicate efforts to resolve how carbon flux is partitioned between primary and secondary pathways. Finally, from a crop perspective, cotton provides classical functional evidence that SUS is required for normal fiber development. More broadly, in non-model crops, available studies already support important roles of SUS in sugar accumulation, sink metabolism, and quality formation, but the overall evidence remains uneven in scope and mechanistic depth across species. Addressing this gap will require more integrative analyses of isoform-specific functions, regulatory networks, and translational applications under agronomically relevant conditions. In particular, integrating natural variation mining, QTL/GWAS analyses, and genome editing in such crop species should improve our understanding of how SUS genes contribute to yield formation, quality traits, and stress adaptation. In conclusion, future SUS research needs to establish tighter connections among molecular mechanisms (including transcriptional, post-transcriptional, and post-translational regulation such as phosphorylation and ubiquitination), spatial organization (organ-specific expression and subcellular localization), whole-plant carbon flux, and population genetics. With ongoing advances in high-resolution imaging, spatial omics, metabolic flux measurements, and integrative multi-omics approaches, a more complete framework for the roles of SUS in plant carbon metabolic networks is likely to emerge, but achieving such a framework will critically depend on systematically designed and rigorously validated experiments across scales.

## Data Availability

Data sharing is not applicable to this article as no new data were generated or analyzed.

## Abbreviations

The following abbreviations are used in this manuscript:

ABA	Abscisic acid
BR	Brassinosteroid
BAR	Bio-analytic resource
CSC	Cellulose synthase complex
DAF	Days after flowering
GA	Gibberellin
qRT-PCR	Quantitative reverse transcription PCR
RLM-RACE	RNA ligase-mediated rapid amplification of cDNA ends
SnRK1	SNF1-related protein kinase 1
SUS	Sucrose synthase
T6P	Trehalose-6-phosphate
UDP	Uridine diphosphate
UDP-Glc	Uridine diphosphate glucose
UGPase	UDP-glucose pyrophosphorylase

## References

[1] Guo S., Li Y., Wang Y., Xu Y., Li Y., Wu P., Wu J., Wang L., Liu X., Chen Z. (2024). OsmiR5519 Regulates Grain Size and Weight and Down-Regulates Sucrose Synthase Gene *RSUS2* in Rice (*Oryza sativa* L.). Planta.

[2] Lastdrager J., Hanson J., Smeekens S. (2014). Sugar Signals and the Control of Plant Growth and Development. J. Exp. Bot..

[3] Mehdi F., Galani S., Wickramasinghe K.P., Zhao P., Lu X., Lin X., Xu C., Liu H., Li X., Liu X. (2024). Current Perspectives on the Regulatory Mechanisms of Sucrose Accumulation in Sugarcane. Heliyon.

[4] Sturm A., Tang G.-Q. (1999). The Sucrose-Cleaving Enzymes of Plants Are Crucial for Development, Growth and Carbon Partitioning. Trends Plant Sci..

[5] Li A.-M., Liao F., Qin C.-X., Wang M., Chen Z.-L., Zhang B.-Q., Gao Y.-J., Pan Y.-Q., Huang D.-L. (2024). Sucrose Phosphate Synthase Genes in Plants: Its Role and Practice for Crop Improvement. J. Agric. Food Chem..

[6] Stein O., Granot D. (2019). An Overview of Sucrose Synthases in Plants. Front. Plant Sci..

[7] Bologa K.L., Fernie A.R., Leisse A., Ehlers Loureiro M., Geigenberger P. (2003). A Bypass of Sucrose Synthase Leads to Low Internal Oxygen and Impaired Metabolic Performance in Growing Potato Tubers. Plant Physiol..

[8] Bai S., Tian Y., Tan C., Bai S., Hao J., Hasi A. (2020). Genome-Wide Identification of microRNAs Involved in the Regulation of Fruit Ripening and Climacteric Stages in Melon (*Cucumis melo*). Hortic. Res..

[9] Hu J., Duan Y., Hu J., Zhang S., Li G. (2024). Phylogenetic and Expression Analysis of the Sucrose Synthase and Sucrose Phosphate Synthase Gene Family in Potatoes. Metabolites.

[10] Mareri L., Guerriero G., Hausman J.-F., Cai G. (2021). Purification and Biochemical Characterization of Sucrose Synthase from the Stem of Nettle (*Urtica dioica* L.). Int. J. Mol. Sci..

[11] Dangwal M., Suri G.S. (2023). Recent Finding on Sucrose Synthase Research: Not the Only Key for Starch and Cellulose Synthesis. Physiol. Mol. Biol. Plants.

[12] Dominguez P.G., Donev E., Derba-Maceluch M., Bünder A., Hedenström M., Tomášková I., Mellerowicz E.J., Niittylä T. (2021). Sucrose Synthase Determines Carbon Allocation in Developing Wood and Alters Carbon Flow at the Whole Tree Level in Aspen. New Phytol..

[13] Fujii S., Hayashi T., Mizuno K. (2010). Sucrose Synthase Is an Integral Component of the Cellulose Synthesis Machinery. Plant Cell Physiol..

[14] Wang C., Jiang H., Gao G., Yang F., Guan J., Qi H. (2023). CmMYB44 Might Interact with CmAPS2-2 to Regulate Starch Metabolism in Oriental Melon Fruit. Plant Physiol. Biochem..

[15] Huang H., Xie S., Xiao Q., Wei B., Zheng L., Wang Y., Cao Y., Zhang X., Long T., Li Y. (2016). Sucrose and ABA Regulate Starch Biosynthesis in Maize through a Novel Transcription Factor, ZmEREB156. Sci. Rep..

[16] Schmölzer K., Gutmann A., Diricks M., Desmet T., Nidetzky B. (2016). Sucrose Synthase: A Unique Glycosyltransferase for Biocatalytic Glycosylation Process Development. Biotechnol. Adv..

[17] Zhao L., Ma Z., Wang Q., Hu M., Zhang J., Chen L., Shi G., Ding Z. (2023). Engineering the Thermostability of Sucrose Synthase by Reshaping the Subunit Interaction Contributes to Efficient UDP-Glucose Production. J. Agric. Food Chem..

[18] Gessler A. (2021). Sucrose Synthase—An Enzyme with a Central Role in the Source–Sink Coordination and Carbon Flow in Trees. New Phytol..

[19] Zhao L., Ma Z., Zhang L., Shen Y., Chen L., Li Y., Xu S., Shi G., Fan D., Ding Z. (2025). Synthesis of Value-Added Uridine 5’-Diphosphate-Glucose from Sucrose Applying an Engineered Sucrose Synthase Counteracts the Activity-Stability Trade-Off. Food Chem..

[20] Decker D., Kleczkowski L.A. (2019). UDP-Sugar Producing Pyrophosphorylases: Distinct and Essential Enzymes With Overlapping Substrate Specificities, Providing de Novo Precursors for Glycosylation Reactions. Front. Plant Sci..

[21] Li J., Hu Y., Hu J., Xie Q., Chen X., Qi X. (2024). Sucrose Synthase: An Enzyme with Multiple Roles in Plant Physiology. J. Plant Physiol..

[22] Wenqi Z. (2025). An Overview of UDP-Glucose Pyrophosphorylase in Plants. Trop. Plant Biol..

[23] Zheng Y., Anderson S., Zhang Y., Garavito R.M. (2011). The Structure of Sucrose Synthase-1 from *Arabidopsis Thaliana* and Its Functional Implications. J. Biol. Chem..

[24] Duncan K.A., Hardin S.C., Huber S.C. (2006). The Three Maize Sucrose Synthase Isoforms Differ in Distribution, Localization, and Phosphorylation. Plant Cell Physiol..

[25] Duncan K.A., Huber S.C. (2007). Sucrose Synthase Oligomerization and F-Actin Association Are Regulated by Sucrose Concentration and Phosphorylation. Plant Cell Physiol..

[26] Yao D., Gonzales-Vigil E., Mansfield S.D. (2020). *Arabidopsis* Sucrose Synthase Localization Indicates a Primary Role in Sucrose Translocation in Phloem. J. Exp. Bot..

[27] Wang W., Viljamaa S., Hodek O., Moritz T., Niittylä T. (2022). Sucrose Synthase Activity Is Not Required for Cellulose Biosynthesis in *Arabidopsis*. Plant J..

[28] Ahmed M., Iqbal A., Latif A., Din S.U., Sarwar M.B., Wang X., Rao A.Q., Husnain T., Ali Shahid A. (2020). Overexpression of a Sucrose Synthase Gene Indirectly Improves Cotton Fiber Quality Through Sucrose Cleavage. Front. Plant Sci..

[29] Liu L., Zheng J. (2022). Identification and Expression Analysis of the Sucrose Synthase Gene Family in Pomegranate (*Punica granatum* L.). PeerJ.

[30] Fan J., Wang H., Li X., Sui X., Zhang Z. (2019). Down-Regulating Cucumber Sucrose Synthase 4 (*CsSUS4*) Suppresses the Growth and Development of Flowers and Fruits. Plant Cell Physiol..

[31] Khanbo S., Somyong S., Phetchawang P., Wirojsirasak W., Ukoskit K., Klomsa-ard P., Pootakham W., Tangphatsornruang S. (2023). A SNP Variation in the Sucrose Synthase (*SoSUS*) Gene Associated with Sugar-Related Traits in Sugarcane. PeerJ.

[32] Song H., Xin J., Yang D., Dong G., Deng X., Liu J., Zhang M., Chen L., Su Y., Yang H. (2024). *NnSUS1* Encodes a Sucrose Synthase Involved in Sugar Accumulation in Lotus Seed Cotyledons. Plant Physiol. Biochem..

[33] Yang T., Huang Y., Liao L., Wang S., Zhang H., Pan J., Huang Y., Li X., Chen D., Liu T. (2024). Sucrose-Associated SnRK1a1-Mediated Phosphorylation of Opaque2 Modulates Endosperm Filling in Maize. Mol. Plant.

[34] Chourey P.S., Taliercio E.W., Carlson S.J., Ruan Y.-L. (1998). Genetic Evidence That the Two Isozymes of Sucrose Synthase Present in Developing Maize Endosperm Are Critical, One for Cell Wall Integrity and the Other for Starch Biosynthesis. Mol. Gen. Genet..

[35] Ruan Y.-L., Llewellyn D.J., Furbank R.T. (2003). Suppression of Sucrose Synthase Gene Expression Represses Cotton Fiber Cell Initiation, Elongation, and Seed Development. Plant Cell.

[36] Bilska-Kos A., Mytych J., Suski S., Magoń J., Ochodzki P., Zebrowski J. (2020). Sucrose Phosphate Synthase (SPS), Sucrose Synthase (SUS) and Their Products in the Leaves of *Miscanthus* × *giganteus* and *Zea mays* at Low Temperature. Planta.

[37] Huang J., Xie B., Xian F., Liu K., Yan Q., He Y., Zhu L., Liu W., Jiang Y., Chen Y. (2025). Gibberellin Signalling Mediates Nucleocytoplasmic Trafficking of Sucrose Synthase 1 to Regulate the Drought Tolerance in Rice. Plant Biotechnol. J..

[38] Li H., Tiwari M., Tang Y., Wang L., Yang S., Long H., Guo J., Wang Y., Wang H., Yang Q. (2022). Metabolomic and Transcriptomic Analyses Reveal That Sucrose Synthase Regulates Maize Pollen Viability under Heat and Drought Stress. Ecotoxicol. Environ. Saf..

[39] Ali M.Y., Chang Q., Yan Q., Qian Z., Guo X., Thow K., Wu J., Zhang Y., Feng Y. (2021). Highly Efficient Biosynthesis of Glycyrrhetinic Acid Glucosides by Coupling of Microbial Glycosyltransferase to Plant Sucrose Synthase. Front. Bioeng. Biotechnol..

[40] Bidart G.N., Hyeuk S., Alter T.B., Yang L., Welner D.H. (2024). A Growth Selection System for Sucrose Synthases (SuSy): Design and Test. AMB Expr..

[41] Chen K., Lin L., Ma R., Ding J., Pan H., Tao Y., Li Y., Jia H. (2023). Identification of Sucrose Synthase from *Micractinium conductrix* to Favor Biocatalytic Glycosylation. Front. Microbiol..

[42] Matera A., Dulak K., Werner H., Sordon S., Huszcza E., Popłoński J. (2024). Investigation on Production and Reaction Conditions of Sucrose Synthase Based Glucosylation Cascade towards Flavonoid Modification. Bioorganic Chem..

[43] Wang Z., Xu Y., Chen T., Zhang H., Yang J., Zhang J. (2015). Abscisic Acid and the Key Enzymes and Genes in Sucrose-to-Starch Conversion in Rice Spikelets in Response to Soil Drying during Grain Filling. Planta.

[44] Liu C., Chen X., Ma P., Zhang S., Zeng C., Jiang X., Wang W. (2018). Ethylene Responsive Factor MeERF72 Negatively Regulates Sucrose Synthase 1 Gene in Cassava. Int. J. Mol. Sci..

[45] Deng Y., Wang J., Zhang Z., Wu Y. (2020). Transactivation of *Sus1* and *Sus2* by Opaque2 Is an Essential Supplement to Sucrose Synthase-mediated Endosperm Filling in Maize. Plant Biotechnol. J..

[46] Niu B., Zhang Z., Zhang J., Zhou Y., Chen C. (2021). The Rice LEC1-like Transcription Factor OsNF-YB9 Interacts with SPK, an Endosperm-specific Sucrose Synthase Protein Kinase, and Functions in Seed Development. Plant J..

[47] Wang Y., Li Y., Cheng F., Zhang S.-P., Zheng Y., Li Y., Li X.-B. (2023). Comparative Phosphoproteomic Analysis Reveals That Phosphorylation of Sucrose Synthase *GhSUS2* by Ca^2+^-Dependent Protein Kinases GhCPK84/93 Affects Cotton Fiber Development. J. Exp. Bot..

[48] Zhang R., An K., Gao Y., Zhang Z., Zhang X., Zhang X., Rossi V., Cao Y., Xiao J., Xin M. (2024). The Transcription Factor CAMTA2 Interacts with the Histone Acetyltransferase GCN5 and Regulates Grain Weight in Wheat. Plant Cell.

[49] Huang T., Zheng T., Hong P., He J., Cheng Y., Yang J., Zhou Y., Wang B., Zhou S., Cheng G. (2025). Sucrose Synthase 3 Improves Fruit Quality in Grape. Plant Physiol. Biochem..

[50] Luo J., Peng F., Zhang S., Xiao Y., Zhang Y. (2020). The Protein Kinase FaSnRK1α Regulates Sucrose Accumulation in Strawberry Fruits. Plant Physiol. Biochem..

[51] Wang Q., Nie X., Luo K., Chen H., Lu M., An H. (2025). PsbHLH58 Positively Regulates Sucrose Accumulation by Modulating Sucrose Synthase 4 in “Fengtang” Plum (*Prunus salicina* Lindl.). Plant Physiol. Biochem..

[52] Sun X., Zhang T., Zhang S., Cui K., Li J. (2025). Transcriptional Regulation and Functional Research of Sucrose Synthase in Plant Development. Planta.

[53] Baud S., Vaultier M.-N., Rochat C. (2004). Structure and Expression Profile of the Sucrose Synthase Multigene Family in *Arabidopsis*. J. Exp. Bot..

[54] Barratt D.H.P., Derbyshire P., Findlay K., Pike M., Wellner N., Lunn J., Feil R., Simpson C., Maule A.J., Smith A.M. (2009). Normal Growth of *Arabidopsis* Requires Cytosolic Invertase but Not Sucrose Synthase. Proc. Natl. Acad. Sci. USA.

[55] Lugassi N., Stein O., Egbaria A., Belausov E., Zemach H., Arad T., Granot D., Carmi N. (2022). Sucrose Synthase and Fructokinase Are Required for Proper Meristematic and Vascular Development. Plants.

[56] Sullivan A., Lombardo M.N., Pasha A., Lau V., Zhuang J.Y., Christendat A., Pereira B., Zhao T., Li Y., Wong R. (2025). 20 Years of the Bio-Analytic Resource for Plant Biology. Nucleic Acids Res..

[57] Liu J., Zhang Y., Zheng Y., Zhu Y., Shi Y., Guan Z., Lang K., Shen D., Huang W., Dou D. (2023). PlantExp: A Platform for Exploration of Gene Expression and Alternative Splicing Based on Public Plant RNA-Seq Samples. Nucleic Acids Res..

[58] Fu Y., Xiao W., Tian L., Guo L., Ma G., Ji C., Huang Y., Wang H., Wu X., Yang T. (2023). Spatial Transcriptomics Uncover Sucrose Post-Phloem Transport during Maize Kernel Development. Nat. Commun..

[59] Piro L., Flütsch S., Santelia D. (2023). *Arabidopsis* Sucrose Synthase 3 (SUS3) Regulates Starch Accumulation in Guard Cells at the End of Day. Plant Signal. Behav..

[60] Xie B., Wang X., Zhu M., Zhang Z., Hong Z. (2011). *CalS7* Encodes a Callose Synthase Responsible for Callose Deposition in the Phloem. Plant J..

[61] Zhang K., Guo L., Cheng W., Liu B., Li W., Wang F., Xu C., Zhao X., Ding Z., Zhang K. (2020). SH1-Dependent Maize Seed Development and Starch Synthesis via Modulating Carbohydrate Flow and Osmotic Potential Balance. BMC Plant Biol..

[62] Hardin S.C., Tang G., Scholz A., Holtgraewe D., Winter H., Huber S.C. (2003). Phosphorylation of Sucrose Synthase at Serine 170: Occurrence and Possible Role as a Signal for Proteolysis. Plant J..

[63] Winter H., Huber J.L., Huber S.C. (1997). Membrane Association of Sucrose Synthase: Changes during the Graviresponse and Possible Control by Protein Phosphorylation. FEBS Lett..

[64] Amor Y., Haigler C.H., Johnson S., Wainscott M., Delmer D.P. (1995). A Membrane-Associated Form of Sucrose Synthase and Its Potential Role in Synthesis of Cellulose and Callose in Plants. Proc. Natl. Acad. Sci. USA.

[65] Haigler C.H., Ivanova-Datcheva M., Hogan P.S., Salnikov V.V., Hwang S., Martin K., Delmer D.P., Carpita N.C., Campbell M., Tierney M. (2001). Carbon Partitioning to Cellulose Synthesis. Plant Cell Walls.

[66] Hardin S.C., Winter H., Huber S.C. (2004). Phosphorylation of the Amino Terminus of Maize Sucrose Synthase in Relation to Membrane Association and Enzyme Activity. Plant Physiol..

[67] Winter H., Huber S.C., Staiger C.J., Baluška F., Volkmann D., Barlow P.W. (2000). Sucrose Metabolism and the Actin Cytoskeleton: SuSy as Actin-Binding Protein. Actin: A Dynamic Framework for Multiple Plant Cell Functions.

[68] Winter H., Huber J.L., Huber S.C. (1998). Identification of Sucrose Synthase as an Actin-Binding Protein. FEBS Lett..

[69] Nunez J.G.A., Kronenberger J., Wuilleme S., Lepiniec L., Rochat C. (2008). Study of *AtSUS2* Localization in Seeds Reveals a Strong Association with Plastids. Plant Cell Physiol..

[70] Buckeridge M.S., Vergara C.E., Carpita N.C. (1999). The Mechanism of Synthesis of a Mixed-Linkage (133),(134)b-D-Glucan in Maize. Evidence for Multiple Sites of Glucosyl Transfer in the Synthase Complex. Plant Physiol..

[71] Parihar P., Jaiswal J.P., Verma A.K., Kumar A. (2025). Sucrose Synthase Dynamics and Its Potential Role in Heat Stress Tolerance in Cereals. Front. Plant Sci..

[72] Li F., Hao C., Yan L., Wu B., Qin X., Lai J., Song Y. (2015). Gene Structure, Phylogeny and Expression Profile of the Sucrose Synthase Gene Family in Cacao (*Theobroma cacao* L.). J. Genet..

[73] Shah I.H., Manzoor M.A., Azam M., Wu J., Li X., Rehman A., Li P., Zhang Y., Niu Q., Chang L. (2025). Comprehensive Characterization and Expression Profiling of Sucrose Phosphate Synthase (SPS) and Sucrose Synthase (SUS) Family in *Cucumis Melo* under the Application of Nitrogen and Potassium. BMC Plant Biol..

[74] Chiu W., Lin C., Chang C., Hsieh M., Wang A. (2006). Molecular Characterization and Expression of Four cDNAs Encoding Sucrose Synthase from Green Bamboo *Bambusa oldhamii*. New Phytol..

[75] Göbel M., Fichtner F. (2023). Functions of Sucrose and Trehalose 6-Phosphate in Controlling Plant Development. J. Plant Physiol..

[76] Mo Y., Jiang B., Huo J., Lu J., Zeng X., Zhou Y., Zhang T., Yang M., Wei Y., Liu K. (2022). Quantitative Ubiquitylomic Analysis of the Dynamic Changes and Extensive Modulation of Ubiquitylation in Papaya During the Fruit Ripening Process. Front. Plant Sci..

[77] Wang M., Du P., Xi L., Lin H., Zhang S. (2026). Dynamic Coordination: How ERF Transcription Factors Coordinate Plant Development and Adaptive Stress Responses. Biomolecules.

[78] Liu Z., Li J., Cao W., Yan Z., Wei W., Gong X., Sun A., Xiao Q. (2026). Transactivation of SuSy1 by SbYABBY8-SbWRKY23 Module Promotes Sucrose Accumulation in Sweet Sorghum Stalks. Ind. Crops Prod..

[79] Hardin S.C., Huber S.C. (2004). Proteasome Activity and the Post-Translational Control of Sucrose Synthase Stability in Maize Leaves. Plant Physiol. Biochem..

[80] Gao X., Li J., Yin J., Zhao Y., Wu Z., Ma L., Zhang B., Zhang H., Huang J. (2024). The Protein Phosphatase qGL3/OsPPKL1 Self-Regulates Its Degradation to Orchestrate Brassinosteroid Signaling in Rice. Plant Commun..

[81] Gao X., Zhang J.-Q., Zhang X., Zhou J., Jiang Z., Huang P., Tang Z., Bao Y., Cheng J., Tang H. (2019). Rice qGL3/OsPPKL1 Functions with the GSK3/SHAGGY-Like Kinase OsGSK3 to Modulate Brassinosteroid Signaling. Plant Cell.

[82] Sun S., Wang T., Wang L., Li X., Jia Y., Liu C., Huang X., Xie W., Wang X. (2018). Natural Selection of a GSK3 Determines Rice Mesocotyl Domestication by Coordinating Strigolactone and Brassinosteroid Signaling. Nat. Commun..

[83] Xiong M., Yu J., Wang J., Gao Q., Huang L., Chen C., Zhang C., Fan X., Zhao D., Liu Q.-Q. (2022). Brassinosteroids Regulate Rice Seed Germination through the BZR1-*RAmy3D* Transcriptional Module. Plant Physiol..

[84] Salnikov V.V., Grimson M.J., Delmer D.P., Haigler C.H. (2001). Sucrose Synthase Localizes to Cellulose Synthesis Sites in Tracheary Elements. Phytochemistry.

[85] Coleman H.D., Ellis D.D., Gilbert M., Mansfield S.D. (2006). Up-regulation of Sucrose Synthase and UDP-glucose Pyrophosphorylase Impacts Plant Growth and Metabolism. Plant Biotechnol. J..

[86] Li Z., Liu S.-L., Montes-Serey C., Walley J.W., Aung K. (2024). PLASMODESMATA-LOCATED PROTEIN 6 Regulates Plasmodesmal Function in *Arabidopsis* Vasculature. Plant Cell.

